# Lasing with cell-endogenous fluorophores: parameters and conditions

**DOI:** 10.1038/s41598-017-12711-x

**Published:** 2017-10-19

**Authors:** Derrick Yong, Ding Ding

**Affiliations:** Precision Measurements Group, Singapore Institute of Manufacturing Technology, 2 Fusionopolis Way, Innovis #08-04, 138634 Singapore, Singapore

## Abstract

The notion of lasing with biologics has recently been realized and has rapidly developed with the collective objective of creating lasers *in vivo*. One major limitation of achieving this is the requirement of exogenous dyes and fluorescent materials. We thus investigate for the first time the possibility of lasing unlabelled cells, using just cell-endogenous fluorophores - the source of cell autofluorescence. In this work, we theoretically studied the lasing potential and efficiency of flavins and reduced nicotinamide adenine dinucleotide (phosphate) (NAD(P)H) using a dye lasing model based on coupled rate equations. Analytical solutions for one- and two-photon pumped system were used in multi-parameter studies. We found that at physiological conditions, the more abundant NAD(P)H can be lased with a cavity quality factor of 10^5^. We then recommended the tuning of intersystem crossing to make the lasing of flavins feasible even at their low physiological concentrations. Under conditions of reduced intersystem crossing, we concluded that it is more practical to lase unlabelled cells using flavins, because lasing thresholds and cavity quality factors were both at least an order lower. We also note the higher threshold requirements and lower efficiencies of two-photon pumping, but recognize its potential for realizing lasing *in vivo*.

## Introduction

Biological lasers (bio-lasers) hold immense potential for applications within biological systems because they are themselves composed of biologics^1^. This concept of generating lasing within or by biologics would be able to circumvent the limited propagation of light in biological tissues as experienced by external laser sources. Since the first demonstration of a single-cell laser^2^ by Gather and Yun in 2011, biological lasers have developed rapidly. This included the demonstration of intracellular lasing using native cell organelles as microcavities^3^ for intracellular sensing as well as by internalizing microresonators^3,4^ for cell tagging and tracking. Aside from cells, bio-lasing has also been demonstrated with biomolecules (flavins^5,6^, green fluorescent protein^7^ and chloropyll^8^) and human tissues (bone^9^ and blood^10^).

Albeit novel and remarkable demonstrations of biological lasers, the generation of lasing by biological cells and tissues still entail the use of externally introduced laser dyes or fluorescent material. Notably, there are biomolecules existing natively within cells that fluoresce. These cell-endogenous fluorophores are the source of autofluorescence, which is often regarded as noise in fluorescence microscopy. They are also the very machineries responsible for cell functions and metabolic activities^11^. These biomolecules have therefore also been employed as endogenous biomarkers for applications like live cell characterization^12,13^ and cell sorting^14,15^. Nevertheless, these are fluorescence emissions and are thus spectrally broad by nature. Such a property makes it difficult to discern between fluorophores with overlapping emission spectra. In contrast, lasing emissions are spectrally narrow and therefore would facilitate the differentation of emissions from several different fluorophores.

In this work, we theoretically determine the feasibility of lasing in unlabelled cells, using just their autofluorescence. Here, we study the conditions and parameters for lasing two of the most abundant cell-endogenous fluorophores - flavins and reduced nicotinamide adenine dinucleotide (phosphate) (NAD(P)H). We do so by using and extending an established organic dye laser model^16^. The framework of the model is based on coupled rate equations that describe the different energy states of our fluorophores. We analytically obtain the lasing thresholds and efficiencies for flavins and NAD(P)H, and identify parameters required for lasing under physiological conditions. We then make recommendations for possible approaches to lower threshold requirements so as to mitigate risks of inducing irreversible cell damage.

## Methods

### Theoretical model

**Figure 1:**
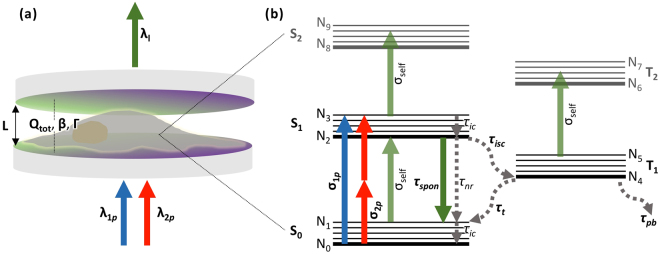
Bio-laser system formed by an unlabelled cell. (**a**) Schematics of an adherent cell within an optical cavity. System is pumped either under a one- (in blue) or two-photon (in red) regime at the wavelengths *λ*_1*p*_ and *λ*_2*p*_ respectively. *λ*_*l*_ is the lasing output (in green) wavelength. The cavity is defined by cavity length (*L*), total quality factor (*Q*_*tot*_), spontaneous emission coupling factor (*β*) and confinement factor of lasing mode (Γ). (**b**) Energy level diagram for cell-endogenous fluorophores. N_0_ to N_9_ are the ten energy levels of a fluorophore. The three lowest singlet states are marked as S_0_, S_1_ and S_2_, while the two lowest triplet states are marked as T_1_ and T_2_. Absorption events are indicated by upward-pointing arrows and tagged with corresponding one-photon (*σ*_1*p*_), two-photon (*σ*_2*p*_) and self- (*σ*_*self*_) absorption cross sections. Radiative and non-radiative relaxation events are denoted by downward-pointing solid and dotted arrows, correspondingly. Relaxation events are appended by their respective lifetimes for spontaneous emission (*τ*_*spon*_), non-radiative relaxation from S_1_ to S_0_ (*τ*_*nr*_), internal conversion (*τ*_*ic*_), intersystem crossing from S_1_ to T_1_ (*τ*_*isc*_), triplet relaxation from T_1_ to S_0_ (*τ*_*t*_) and photobleaching (*τ*_*pb*_).

In this study, we investigated the parameters and conditions for lasing in biological cells without the introduction of exogenous laser dyes or fluorescent materials. We modelled the bio-laser construct as an adherent cell within an optical cavity, pumped by an external pulsed laser source as illustrated in Fig. 1a. The endogenous fluorophores, flavins and NAD(P)H, were analyzed for their potential as laser gain media under physiological conditions. It should be noted that flavins here refer to all three fluorescent derivatives found natively within cells, namely riboflavins (RF), flavin mononucleotides (FMN) and flavin adenine dinucleotide (FAD); while NAD(P)H collectively refers to the fluorescent reduced forms of nicotinamide adenine dinucleotide (NAD) and nicotinamide adenine dinucleotide phosphate (NADP). The parameters that define each endogenous fluorophore are listed in Table 1. In the model, we assumed a homogenous distribution of either endogenous fluorophore within a 100 *μ*m × 100 *μ*m × 5 *μ*m volume. These dimensions correspond to the approximate dimensions of an adherent cell stretched over a 100 *μ*m × 100 *μ*m area with a height of 5 *μ*m - equivalent to the cavity length. We also assumed a stable optical cavity configuration, where light is confined within the gain region based on the study^17^ by Humar *et al*.

**Table 1:** Parameters used in one- and two-photon pumped lasing models.

Parameters	Flavins	NAD(P)H
Pump area, *A*	1 × 10^−4^ cm^2^	
Cavity length, *L*	5 *μ*m	
Quality factor of cavity due to radiative loss, *Q*_*rad*_	1 × 10^5^	
Spontaneous emission coupling factor, *β*	1 × 10^−4^ ^36^ ^,*a*^	
Confinement factor of lasing mode, Γ	0.2	
One-photon pump wavelength, *λ*_1*p*_	390 nm	
Two-photon pump wavelength, *λ*_2*p*_	780 nm	
Lasing output wavelength, *λ*_*l*_	580 nm	500 nm
One-photon absorption cross section, *σ*_1*p*_(*λ*_1*p*_)	3.3 × 10^−17^ cm^2^ ^37^	2.0 × 10^−18^ cm^2^ ^38^
Two-photon absorption cross section, *σ*_2*p*_(*λ*_2*p*_)	7.8 × 10^−33^ cm^4^ W^−1^ ^39^	3.9 × 10^−35^ cm^4^ W^−1^ ^39^
Self-absorption cross section of output *S*_0_ → *S*_1_, ${\sigma }_{self}^{{S}_{0}{S}_{1}}$	10^−20^ cm^2^	10^−21^ cm^2^
Self-absorption cross section of output *S*_1_ → *S*_2_, ${\sigma }_{self}^{{S}_{1}{S}_{2}}$	10^−18^ cm^2^	10^−19^ cm^2^
Self-absorption cross section of output *T*_1_ → *T*_2_, ${\sigma }_{self}^{{T}_{1}{T}_{2}}$	10^−18^ cm^2^	10^−19^ cm^2^
Fluorescence quantum yield, *ϕ*_*F*_	0.26^27,40,41^	0.019^42^
Spontaneous emission lifetime, *τ*_*spon*_	4.6 ns^27–29^	0.4^21,43–45^
Internal conversion lifetime, *τ*_*ic*_	1 ps	1 ps
Intersystem crossing lifetime, *τ*_*isc*_	13.6 ns^26^	~10^5^ ^30^
Triplet decay lifetime, *τ*_*t*_	27 *μ*s^34^	2.7 s^35^
Intracellular concentration, *C*	~10^−6^ M^20^ ^,*b*^	~10^−5^ M^21^
Critical transfer concentration, *C*_0_	4.7 × 10^−2^ M^46^	3.5 × 10^−4^ M^47^
Dimerization constant, *K*_*D*_	118 M^−1^ ^48^	NA^49,50^ ^,*c*^

^*a*^Estimated based on the linewidth ratio between spontaneous emission and the lasing mode; ^*b*^Based on total flavin content per cell (i.e. combination of RF, FMN and FAD) and cell volume of 10^−15^ m^3^; ^*c*^No observations of NAD(P)H dimerization were reported, only electrochemically generated dimers of its non-fluorescent oxidized form (NAD(P)).

The theoretical framework in this study is based on eleven coupled rate equations that describe ten energy levels (N_0_ to N_9_) and an output as depicted by the energy level diagram in Fig. 1. These rate equations are based on an established organic dye laser model^16^. To incorporate the two-photon pumping regime, the terms describing the rate of pumping were reformulated. In the one-photon regime it takes the form:

1 $$rat{e}_{1p}=\frac{{I}_{pump}A}{h{f}_{1p}}\times (1-{10}^{-{\sigma }_{1p}{N}_{den}L})$$

where *I*_*pump*_ is the input pump intensity; *h* is Planck’s constant; *f*_1*p*_ is the frequency of the input pump under one-photon pumping; *σ*_2*p*_ is the one-photon absorption cross section; *N*_*den*_ is the number density of the fluorophore, calculated from *N*_*A*_ *C*/1000 (*N*_*A*_: Avogadro’s constant; *C*: molar concentration); A is the area of pumping; and L is the thickness of the cavity. While in the two-photon regime, the rate of pumping takes the form:

2 $$\begin{array}{rcl}rat{e}_{2p} & = & \frac{{I}_{pump}^{2}A}{2h{f}_{2p}}\times \frac{{\sigma }_{2p}{N}_{den}L}{1+{I}_{pump}{\sigma }_{2p}{N}_{den}L}\\  & \approx  & \frac{{I}_{pump}^{2}A}{2h{f}_{2p}}\times {\sigma }_{2p}{N}_{den}L\end{array}$$

where *f*_2*p*_ is the frequency of the input pump under two-photon pumping and *σ*_2*p*_ is its corresponding two-photon absorption cross section. Note that the approximation holds in this study across all considered fluorophore concentrations. Under the most ideal case of two-photon absorption by flavins at a concentration of 0.1 M, deviation only occurs when pump intensities exceed 10^17^ W cm^−2^. All coupled rate equations were solved numerically in MATLAB. Parameters used in computations are listed in Table 1.

### Lasing threshold

Simplified analytical solutions to lasing thresholds were derived from the couple rate equations at steady state (i.e. *dN*_*i*_/*dt* = 0, where *N*_*i*_ corresponds to the different energy levels). The total fluorophore population is assumed to be N_*tot*_ = N_0_ + N_2_ + N_4_. This assumption is valid when: (i) pulsed excitation is considered where the rate of photobleaching (1/*τ*_*pb*_) is orders of magnitudes slower; (ii) pump absorption is negligible for S_1_ to S_2_ and T_1_ to T_2_ transitions; (iii) pump intensities are reasonably low, such that other levels are negligibly populated. The one-photon pumped lasing threshold^16^ is:

3 $${I}_{thres\mathrm{,1}p}=\frac{h{f}_{1p}}{{\sigma }_{1p}{N}_{den}}\times \frac{(\frac{1}{{\varphi }_{F}{\tau }_{spon}}+\frac{1}{{\tau }_{isc}})/{\tau }_{loss}^{{S}_{0}{S}_{1}}}{\frac{\beta {\rm{\Gamma }}V}{{\tau }_{spon}}-(1+\frac{{\tau }_{t}}{{\tau }_{isc}})/{\tau }_{loss}^{{S}_{1}{S}_{2}}{N}_{den}}$$

where *ϕ*_*F*_ is the fluorescence quantum yield; *τ*_*spon*_, *τ*_*isc*_ and *τ*_*t*_ correspond to the lifetimes of spontaneous emission, intersystem crossing from S_1_ to T_1_ and triplet relaxation from T_1_ to S_0_ respectively; *β* is the spon-taneous emission coupling factor; Γ is the confinement factor of the lasing mode; *V* is the gain volume defined by *V* = *AL*; ${\tau }_{loss}^{{S}_{0}{S}_{1}}$ and ${\tau }_{loss}^{{S}_{1}{S}_{2}}$ are the combined losses from the passive cavity’s photon decay lifetime (*τ*_*loss*_) and self-absorption of the output from S_0_ to S_1_ and S_1_ to S_2_ (or T_1_ to T_2_) respectively. $({\tau }_{loss}^{{S}_{0}{S}_{1}}={(1/{\tau }_{loss}+{v}_{g}{\sigma }_{self}^{{S}_{0}{S}_{1}}{\rm{\Gamma }}{N}_{den})}^{-1}$ and ${\tau }_{loss}^{{S}_{1}{S}_{2}}={(1/{\tau }_{loss}+{v}_{g}{\sigma }_{self}^{{S}_{1}{S}_{2}}{\rm{\Gamma }}{N}_{den})}^{-1}$, where *τ*_*loss*_ = *Q*_*tot*_/2*πf*_*l*_; *f*_*l*_ is the lasing frequency; *v*_*g*_ is the group velocity of the lasing output; *σ*_*self*_ is the self-absorption cross section). Similarly, we derived the lasing threshold under two-photon pumping as:

4 $${I}_{thres\mathrm{,2}p}=\sqrt{\frac{2h{f}_{2p}}{{\sigma }_{2p}{N}_{den}}\times \frac{(\frac{1}{{\varphi }_{F}{\tau }_{spon}}+\frac{1}{{\tau }_{isc}})/{\tau }_{loss}^{{S}_{0}{S}_{1}}}{\frac{\beta {\rm{\Gamma }}V}{{\tau }_{spon}}-(1+\frac{{\tau }_{t}}{{\tau }_{isc}})/{\tau }_{loss}^{{S}_{1}{S}_{2}}{N}_{den}}}$$

Effects of concentration quenching by dimerization^18,19^ on *ϕ*_*F*_ and *τ*_*spon*_ were also included in the lasing threshold analysis using the parameters acquired for the fluorophores’ critical transfer concentration (*C*_0_) and dimerization constant (*K*_*D*_).

### Lasing efficiency

Simplified analytical solutions to lasing efficiencies were likewise derived based on the same assumptions. The one-photon pumped lasing efficiency^16^ is:

5 $${q}_{lase\mathrm{,1}p}=\frac{{f}_{l}}{{f}_{1p}}\times \frac{(1-{10}^{-{\sigma }_{1p}{N}_{den}L})}{{\tau }_{cav}\Gamma }\times \frac{\frac{\beta {\rm{\Gamma }}V}{{\tau }_{spon}}-(1+\frac{{\tau }_{t}}{{\tau }_{isc}})/{\tau }_{loss}^{{S}_{1}{S}_{2}}{N}_{den}}{\frac{\beta V}{{\tau }_{spon}{\tau }_{loss}}+{v}_{g}{\sigma }_{self}^{{S}_{0}{S}_{1}}[\frac{1}{{\tau }_{loss}}+(1+\frac{{\tau }_{t}}{{\tau }_{isc}})/{\tau }_{loss}^{{S}_{1}{S}_{2}}]}$$

*q*_*lase*,1*p*_ is essentially the gradient of the post-threshold linear slope for the input-output intensity plot (i.e. *q*_*lase*,1*p*_ = *dI*_*out*_/*dI*_*pump*_). Here, *τ*_*cav*_ is the photon decay lifetime due to radiative loss from the cavity. We also derive the two-photon pumped lasing efficiency as:

6 $${q}_{lase\mathrm{,2}p}=\frac{{f}_{l}}{2{f}_{2p}}\times \frac{{\sigma }_{2p}{N}_{den}L}{{\tau }_{cav}{\rm{\Gamma }}}\times \frac{\frac{\beta {\rm{\Gamma }}V}{{\tau }_{spon}}-(1+\frac{{\tau }_{t}}{{\tau }_{isc}})/{\tau }_{loss}^{{S}_{1}{S}_{2}}{N}_{den}}{\frac{\beta V}{{\tau }_{spon}{\tau }_{loss}}+{v}_{g}{\sigma }_{self}^{{S}_{0}{S}_{1}}[\frac{1}{{\tau }_{loss}}+(1+\frac{{\tau }_{t}}{{\tau }_{isc}})/{\tau }_{loss}^{{S}_{1}{S}_{2}}]}$$

where ${q}_{lase\mathrm{,2}p}=d{I}_{out}/d{I}_{pump}^{2}$. Here, *q*_*lase*,2*p*_ has been formulated as a dimensionless term like *q*_*lase*,1*p*_.

## Results

### Lasing thresholds

**Figure 2:**
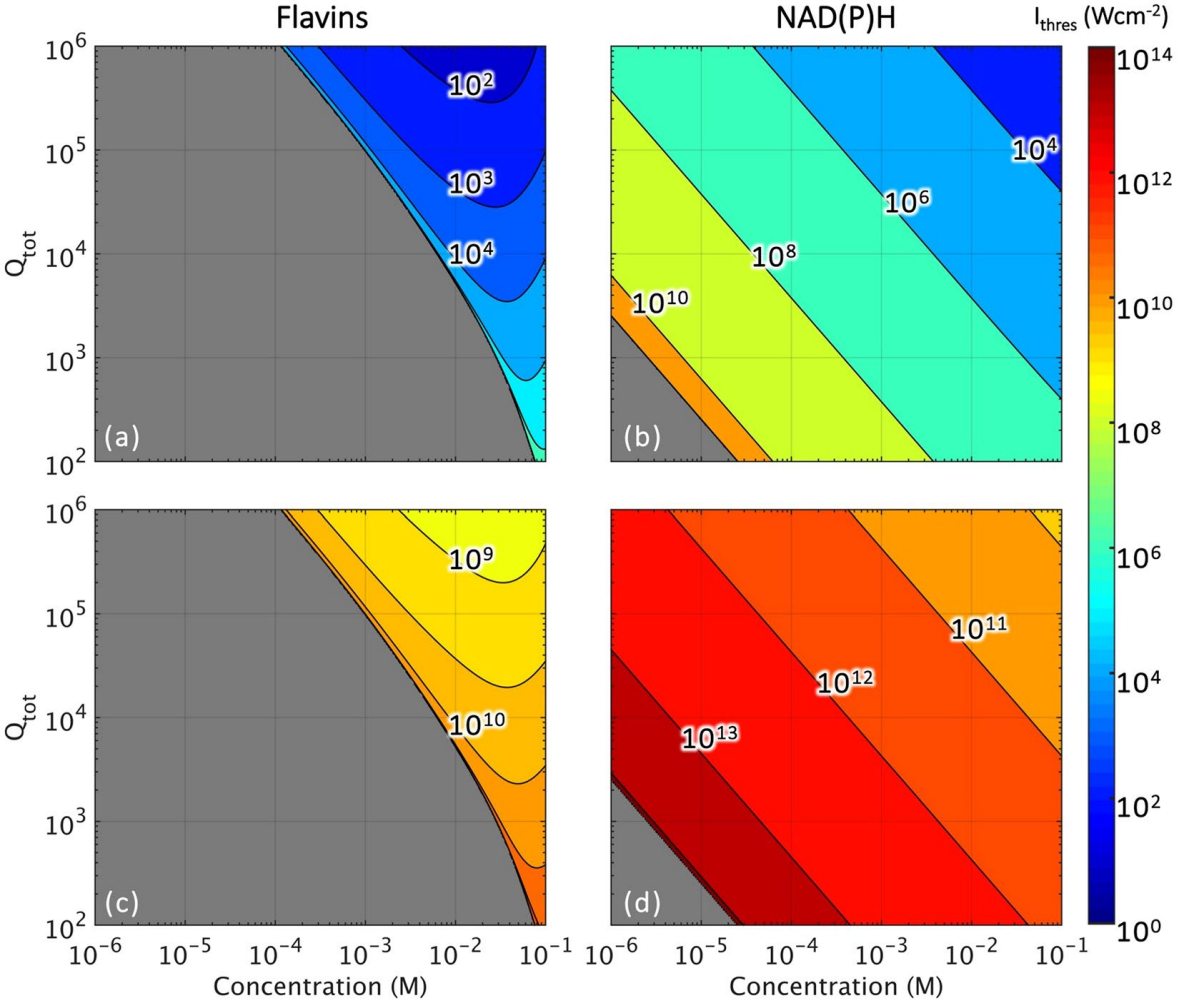
Lasing threshold intensities (in W cm^−2^) as a function of total quality factor of the cavity (*Q*_*tot*_) and fluorophore concentration (*C*) under (**a**,**b**) one- and (**c**,**d**) two-photon photon pumping for (**a**,**c**) flavins and (**b**,**d**) NAD(P)H. It should be noted that threshold intensity values have been plot based on their order of magnitude. Regions in grey indicate parameters that do not support lasing.

In this section, we studied the order of pump intensities required for lasing by varying 3 parameters - total quality factor of cavity (*Q*_*tot*_), concentration of fluorophores (*C*) and ratio of lifetimes for intersystem crossing (*τ*_*t*_/*τ*_*isc*_). Lasing thresholds were computed using Equations 1 and 2. In Fig. 2, lasing thresholds are reported as a function of *Q*_*tot*_ and *C* and we note two key observations from the plots. First, NAD(P)H supports lasing over a wider range of concentrations as compared to flavins where lasing cannot be achieved below 10^−4^ M. This is true under both one- and two-photon pumping. Next, we note higher threshold intensities for NAD(P)H. Threshold intensities were computed to range from 10^2^ to 10^11^ W cm^−2^ for NAD(P)H and 10^1^ to 10^6^ W cm^−2^ for flavins under one-photon pumping. For two-photon pumping, threshold intensities ranged from 10^9^ to 10^14^ W cm^−2^ for NAD(P)H and 10^8^ to 10^11^ W cm^−2^ for flavins.

**Figure 3:**
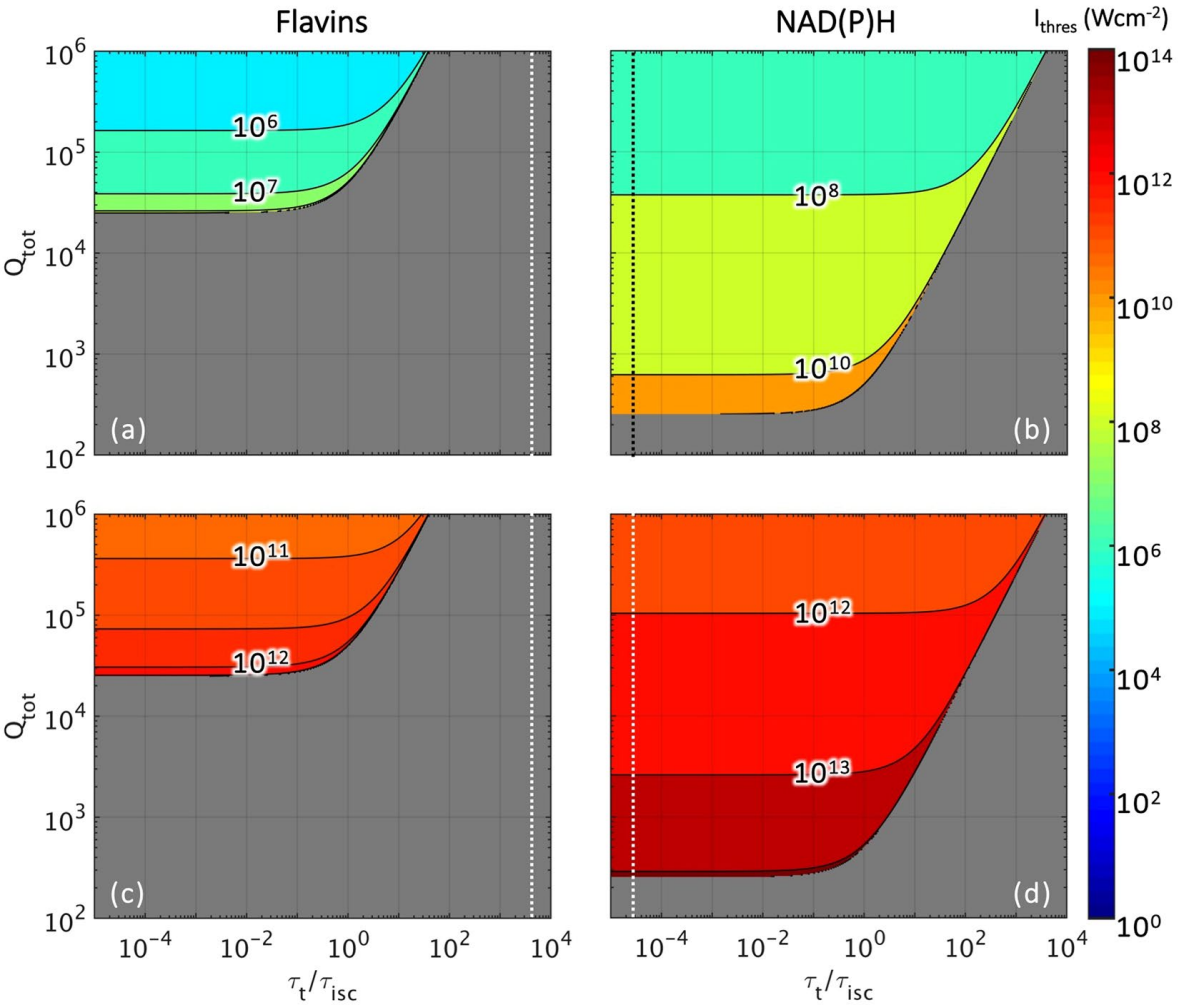
Lasing threshold intensities (in W cm^−2^) as a function of total quality factor of the cavity (*Q*_*tot*_) and ratio of lifetimes for intersystem crossing (*τ*_*t*_/*τ*_*isc*_) under (**a**,**b**) one- and (**c**,**d**) two-photon photon pumping for (**a**,**c**) 10^−6^ M flavins and (**b**,**d**) 10^−5^ M NAD(P)H. It should be noted that threshold intensity values have been plot based on their order of magnitude. Regions in grey indicate parameters that do not support lasing. Dotted lines denote *τ*_*t*_/*τ*_*isc*_ at physiological conditions (~2 × 10^3^ for flavins^26,34^ and ~3 × 10^−5^ for NAD(P)H^30,35^).

In Fig. 3, we study the effects of varying *Q*_*tot*_ and *τ*_*t*_/*τ*_*isc*_ on lasing thresholds. Threshold intensities were computed for intracellular concentrations of flavins^20^ and NAD(P)H^21^ at ~10^−6^ M and ~10^−5^ M respectively. Here, we note that lasing is supported in NAD(P)H at lower values of *Q*_*tot*_. Under both pumping regimes, lasing can be achieved from *Q*_*tot*_ of 2 × 10^2^ for NAD(P)H and 2 × 10^4^ for flavins. In addition, we highlight that at physiological conditions of *τ*_*t*_/*τ*_*isc*_, lasing is not supported by flavins. This is indicated by the white-dotted line residing within the non-lasing region of the Fig. 3a,c.

We also computed lasing threshold intensities for green fluorescent protein (GFP) in cells based on parameters detailed in the work by Gather and Yun on the first single-cell laser^2^. Parameters included a gain length (L) and a pump area (A) calculated from the 15 *μ*m diameter of a cell as well as gain properties of GFP. At the desribed experimental conditions, where intracellular GFP concentration was ~300 *μ*M and *Q*_*tot*_ was >10^4^ (estimated from the reported lasing wavelength of 516 nm with a full width at half maximum of <0.04 nm), we obtained a lasing threshold intensity of ~63 kW cm^−2^. At a pulse duration of 5 ns, this converts to a lasing threshold pulse energy of ~0.56 nJ, which concurs well with their experimentally obtained threshold of ~1 nJ. We attribute the higher energy noted in experiments to the actual pump area extending beyond the cross sectional area of the cell.

### Lasing efficiencies

We further analyzed the efficiency of lasing for 10^−5^ M NAD(P)H for different *Q*_*tot*_. Lasing efficiency corresponds to the amount of output lasing intensity generated per unit of input pump intensity (or square of input pump intensity for the two-photon regime). Efficiencies were computed using Equations 5 and 6 and are reported in Table 2. For both regimes of pumping, efficiencies are noted to decrease by approximately an order per order of decrease in *Q*_*tot*_. We further observe that efficiencies under one-photon pumping are 18 to 19 orders higher than their two-photon pumped counterparts.

**Table 2:** Lasing efficiencies for 1 × 10^−5^ M NAD(P)H under one- and two-photon pumping in systems with different total cavity quality factor (*Q*_*tot*_).

	*Q*_*tot*_		
	1 × 10^5^	1 × 10^4^	1 × 10^3^
*q*_*lase*,1*p*_	1.05 × 10^−5^	1.03 × 10^−6^	7.88 × 10^−8^
*q*_*lase*,2*p*_	9.20 × 10^−23^	8.97 × 10^−24^	6.91 × 10^−25^

Next, we examined the deviation of simplified analytical solution from numerically computed results. Initial computations revealed significant deviations from the numerical solution especially at low *Q*_*tot*_. At high pump intensities, assumption (iii) that was stated in the derivation of lasing threshold solutions becomes invalid. A semi-simplified analytical solution was thus derived by considering N_*tot*_ = N_0_ + N_2_ + N_3_ + N_4_. This solution under one-photon pumping was derived as follows. First we obtained expressions to eliminate terms defining the populations at different energy levels:

7 $${F}_{30}=\frac{{N}_{3}}{{N}_{0}}=\frac{\frac{{I}_{pump}A}{h{f}_{1p}{N}_{tot}}(1-{10}^{-{\sigma }_{1p}{N}_{den}L})}{\frac{1}{{\tau }_{ic}}+\frac{{I}_{pump}A}{h{f}_{1p}{N}_{tot}}(1-{10}^{-{\sigma }_{1p}{N}_{den}L})}$$

8 $${F}_{02}=\frac{{N}_{0}}{{N}_{2}}=\frac{\frac{{I}_{out}A}{h{f}_{l}}({v}_{g}{\sigma }_{self}^{{S}_{0}{S}_{1}}{\rm{\Gamma }}+\frac{\beta {\rm{\Gamma }}}{{\tau }_{spon}})+(\frac{1}{{\varphi }_{F}{\tau }_{spon}}+\frac{1}{{\tau }_{isc}})}{\frac{{I}_{pump}A}{h{f}_{1p}{N}_{tot}}(1-{10}^{-{\sigma }_{1p}{N}_{den}L})\,\mathrm{(1}-{F}_{30})+\frac{{I}_{out}A}{h{f}_{l}V}({v}_{g}{\sigma }_{self}^{{S}_{0}{S}_{1}}{\rm{\Gamma }})}$$

The above expressions were then incorporated into the following equation:

9 $$\tfrac{{I}_{pump}A}{h{f}_{1p}}\times (1-{10}^{-{\sigma }_{1p}{N}_{den}L})\times \tfrac{{F}_{02}\mathrm{(1}-{F}_{30})}{{F}_{02}\mathrm{(1}+{F}_{30})+(1+\tfrac{{\tau }_{t}}{{\tau }_{isc}})}=\tfrac{{N}_{tot}(\tfrac{1}{{\varphi }_{F}{\tau }_{spon}}+\tfrac{1}{{\tau }_{isc}})}{{F}_{02}\mathrm{(1}+{F}_{30})+(1+\tfrac{{\tau }_{t}}{{\tau }_{isc}})}+\tfrac{{I}_{out}A}{h{f}_{l}{\tau }_{loss}}$$

A semi-simplified analytical solution for I_*out*_ was then solved from the above using MATLAB. The same was done for the two-photon regime, by replacing the rate of pumping terms from what is defined in Equation 3 to that in Equation 4.

**Figure 4:**
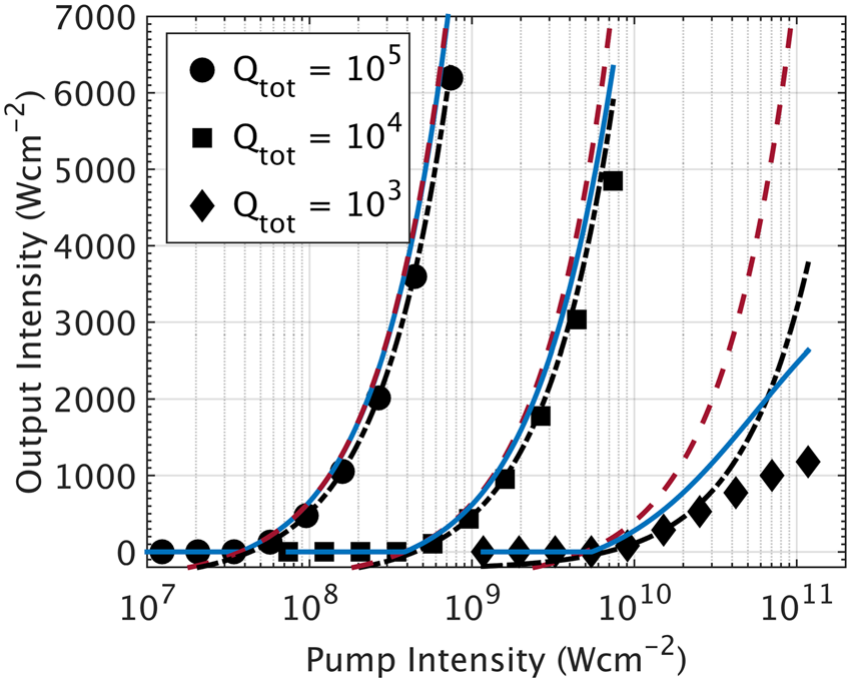
Output-input intensity plots for one-photon pumped 1 × 10^−5^ M NADH in systems with different total cavity quality factor (*Q*_*tot*_). Numerically computed data are represented by solid black circles, squares and diamonds for *Q*_*tot*_ of 10^5^, 10^4^ and 10^3^ respectively. Black dash-dotted lines are linear fits of the first two numerical data points post-lasing threshold. Red dashed lines are plots obtained from the simplified analytical solutions to lasing threshold (*I*_*thres*,1*p*_) and efficiency (*q*_*lase*,1*p*_). Blue lines are semi-simplified analytical solutions to the coupled rate equations.

In Fig. 4, we see the differences between the simplified (red dashed lines) and semi-simplified analytical solutions (blue solid lines) and the numerical data (black-coloured data points). This difference is observed to increase with decreasing *Q*_*tot*_. This difference is further quantified by taking the difference between analytical and numerical solutions as a percentage of the corresponding numerical solutions. We report these calculated percentage deviations for lasing efficiency in Table 3. To obtain lasing efficiencies from the numerical solution, linear fits were made with the first two data points post-lasing threshold. The corresponding range of data points for the semi-simplified analytical solution were then used in obtaining their respective lasing efficiencies.

**Table 3:** Percentage deviation of analytically-obtained lasing efficiencies from numerical data for one-photon pumped 1 × 10^−5^ M NAD(P)H in systems with different total cavity quality factor (*Q*_*tot*_).

	*Q*_*tot*_		
	1 × 10^5^	1 × 10^4^	1 × 10^3^
*q*_*lase*,1*p*_ from simplified analytical solution	15.7%	21.4%	132%
Linear fit of semi-simplified analytical solution	15.4%	18.2%	58.5%

## Discussion

### Lasing at physiological conditions

At physiological conditions, where intracellular concentrations of flavins and NAD(P)H are at ~10^−6^ M and ~10^−5^ M respectively, we note from Fig. 2 that it is not possible to lase flavins. It is however possible to lase NAD(P)H with high pump intensities. According to the ICNIRP guidelines^22^, the damage threshold for tissues (skin) exposed to visible lasers of nanosecond pulses is 20 mJ cm^−2^ - for a 1 ns pulse, this is equivalent to 20 MW cm^−2^. From Fig. 2b, we identify a required total cavity quality factor of *Q*_*tot*_ > 10^5^ to lase NAD(P)H without inducing damage. Here, we would like to highlight that *Q*_*tot*_ is a collective term that comprises both extrinsic (radiative decay) and intrinsic components. The latter consists of the quality factor of the passive cavity (*Q*_*cav*_) and material-induced losses that includes absorption and scattering losses induced by a cell. These imply that the *Q*_*cav*_ required is higher than *Q*_*tot*_. Nevertheless, based on the experimental demonstrations of cell lasers, we note that *Q*_*cav*_ does not differ by more than an order from *Q*_*tot*_ − 1.6 × 10^4^ versus 1.3 × 10^4^ in the work by Gather and Yun on a single-cell laser^2^; and 1.8 × 10^4^ versus 2.1 × 10^3^ in the work by Humar and Yun on an intracellular microlaser^3^. Total quality factors of >10^5^ are thus still attainable by whispering gallery mode (WGM) microresonators^23^ and even Fabry-Pérot microcavities that enable three-dimensional confinement^24^. However, considering that a WGM microresonator requires the fluorophores to reside at its periphery to effectively generate lasing, it becomes a challenge for microresonators in a cell to be able to tap on all of the intracellular fluorophores. Fabry-Pérot cavities, on the contrary, offer a more efficient means of utilizing all of the cell’s contents since the fluorophore need only be sandwiched between a pair of mirrors. On the other hand, when pumped at NIR wavelengths, the ICNIRP guidelines do not state any thresholds for sub-nanosecond pulses for irradiation of skin tissue, instead it only suggests a conservative damage threshold of ~20 GW cm^−2^ for NIR femtosecond pulses. The guidelines do however list a damage threshold of 1 MW cm^−2^ for exposure of the eye to 100 fs NIR pulses. A study^25^ on cells in culture irradiated by multiple (76 MHz over 0.25 s) 130 fs NIR (810 nm) pulses at a beam diameter of ~100 *μ*m reports a damage threshold of 1.9 kJ cm^−2^. By taking the very conservative assumption that damage is induced upon irradiation by the very first pulse (at 0.1 mJ cm^−2^), we estimate a threshold intensity of ~1 GW cm^−2^. From Fig. 2d, we see that lasing NAD(P)H is possible at physiological conditions under two-photon pumping by NIR lasers, but the reported damage thresholds imply the cell’s inevitable demise. It is hence more feasible to lase NAD(P)H in cells under the one-photon regime than by its two-photon counterpart.

It is interesting to note that although NAD(P)H have both absorption cross sections and quantum yields at an order of magnitude smaller than flavins, they are able to support a much larger range of conditions for lasing. This is counterintuitive considering that flavins are able to absorb and emit more efficiently than NAD(P)H. From the energy level diagram in Fig. 1b, we understand this to be due to intersystem crossing from the singlet (S_1_) to triplet (T_1_) state. In flavins, the intersystem crossing lifetime^26^ (*τ*_*isc*_) is of the same nanosecond timescale as its spontaneous emission lifetime^27–29^ (*τ*_*spon*_). This implies competition between transitions from the upper laser level (N_2_) to either the lower laser level (N_1_) or the lower triplet level (N_4_). Furthermore, the decay rate of flavins from its triplet to singlet state is three orders slower than its rate of triplet formation, resulting in the “trapping” of excited fluorophores in a energy state not usable in lasing transitions. In comparison, NAD(P)H has virtually negligible triplet transitions^30^. From Fig. 3a,c, we observe again that at physiological conditions (denoted by vertical dotted lines) lasing is not supported by flavins. From the plots, we also see that reducing *τ*_*t*_/*τ*_*isc*_ to a value of ~10 would render flavins capable of lasing at its physiological intracellular concentration with Q_*tot*_ < 10^6^. This can be achieved by reducing *τ*_*t*_ and increasing *τ*_*isc*_, which translates to faster decays from the triplet state and reduced intersystem crossings respectively. The use of iodide as quenchers has been reported to reduce the triplet population and increase fluorescence in flavins when used in small quantities^31^. Although iodide is known to increase the rate of intersystem crossing (results in higher triplet populations), the authors have attributed their findings to the concomitant increase in triplet decay rates, which undergo a relatively higher increment than that of intersystem crossing. Alternatively, binding of flavins with light-oxygen-voltage-sensing (LOV) proteins has also been demonstrated to reduce the rate of intersystem crossing^26^, again resulting in a lower triplet population. This interaction with LOV proteins was reported to reduce *τ*_*t*_ by an order, which similarly reduces *τ*_*t*_/*τ*_*isc*_ by an order from ~2,000 to <200. From our calculations, this would support lasing when *Q*_*tot*_ is ~4 × 10^6^ and would only require pump intensities of a few MW cm^−2^ - well within the damage threshold. Since mechanisms for tuning *τ*_*t*_ and *τ*_*isc*_ exist, and flavins require lasing threshold intensities that are several-orders lower than NAD(P)H, we find it more practical to achieve lasing in unlabelled cells using flavins. This is provided that the condition of *τ*_*t*_/*τ*_*isc*_ < 1 can be achieved, which would allow lasing of flavins at physiological intracellular concentrations with just a *Q*_*tot*_ of ~4 × 10^4^ and without exceeding the damage threshold. Such a *Q*_*tot*_ is still attainable under the most ideal conditions of a Fabry-Pérot cavity comprising a mirror pair.

### One- vs. two-photon pumping

When we compare one- and two-photon pumping, we note two keys points for discussion. First, threshold intensity requirements are many orders of magnitude higher for the two-photon regime. This is consequent of the nature of multi-photon processes, where two or more photons have to arrive simultaneously for energy transitions to take place. Such a requirement translates into a large number of photons arriving over a short period of time in a small area, ergo high intensities (in W cm^−2^). That being said, it should be highlighted that such a property is in fact an advantage of multi-photon processes, particularly in the biosciences^32^. The high intensity requirements enable spatial specificity and transparency of materials at low intensities. Although not considered in this study, this property could enable the lasing of specific cells buried within tissues provided that cavity requirements can be met intracellularly. Furthermore, the typical use of NIR wavelengths in such process also enables deeper penetration *in vivo* ^33^, indicating the possibility of *in vivo* laser generation. Similarly, as would be expected, lasing efficiencies are many orders of magnitude lower considering the high intensities already required to first excite the fluorophores. Secondly, we note the parameters that do not support lasing are dependent only on the fluorophore and not the regime of pumping. This is seen in the identical grey regions shaded in Figs 2 and 3. From Equations 3 and 4, lasing is not supported when the denominator of the last collection of terms becomes negative. This occurs when $\beta {\rm{\Gamma }}V/{\tau }_{spon} < \mathrm{(1}+{\tau }_{t}/{\tau }_{isc})/{\tau }_{loss}^{{S}_{1}{S}_{2}}{N}_{den}$ (refer to Methods and Table 1 for details) - i.e. when the maximal available gain is smaller than the intrinsic losses of the system. This is thus independent on the pump but still dependent on cavity properties, namely the spontaneous emission coupling factor (*β*) and lasing mode confinement (Γ). However, should the spatial specificity of multi-photon processes be considered, the pump area effectively changes and consequently the available fluorophores within said area. It is thus particularly important to note the beam diameter when employing the two-photon regime described in this study.

### Saturation of higher excited state (N_3_)

At high pump intensities we observe a deviation the simplified analytical solution from its numerical counterpart. We attribute this to the breakdown of the assumption that only N_0_, N_2_ and N_4_ are populated. When pumped at high intensities, the rate of internal conversion from N_3_ to N_2_ (1/*τ*_*ic*_ = 1 × 10^12^ *s* ^−1^) becomes insufficient to significantly depopulate N_3_. This results in saturation of the fluorophore population in this higher excited state, which is different from the typical saturation we observe in two-level laser systems. For 10^−5^ M NAD(P)H, the rate of excitation from N_0_ to N_3_ matches the rate of depopulation from N_3_ to N_2_ when pump intensities exceed 10^4^ W cm^−2^ and 10^10^ W cm^−2^ under one- and two-photon pumping, correspondingly. From threshold intensities in Fig. 2, we note that this applies to all ranges of parameters studied. Hence, non-negligible populations of fluorophores exist in N_3_. A semi-simplified analytical solution was hence derived by considering N_*tot*_ = N_0_ + N_2_ + N_3_ + N_4_. We compared the simplified analytical, semi-simplified analytical and numerical solutions in Fig. 4 and further quantified deviations of the analytical solutions from the numerical solution in Table 3. It should be noted that the x-axis of the input-output plot is in the log scale, which accounts for the non-linear curves. We first observe that all lasing thresholds were well within the same order of magnitude, implying no need to derive more complex solutions for threshold computations. Lasing efficiencies, on the other hand, are observed to differ significantly in Fig. 4, especially for a low *Q*_*tot*_ of 10^3^. We also see how the semi-simplified analytical solution better matches the numerical solution, with its relative amount of deviation improving for lower values of *Q*_*tot*_. In that regard, we recommend the use of the semi-simplified analytical solution when studying poorly performing cavities so as to not get an over estimate of lasing efficiency.

## Conclusion

In summary, we have theoretically studied the feasibility of lasing with cell-endogenous fluorophores and identified parameters that allow for lasing in cells at physiological conditions. We found that lasing is supported by NAD(P)H but not flavins at physiological intracellular concentrations. This could be achieved using cavities with *Q*_*tot*_ > 10^5^ under the one-photon pumping regime. From further analysis of the fluorophores’s transitions, we identified the intersystem crossing in flavins to be a key reason for its inability to support lasing. We then made recommendations to tune *τ*_*t*_/*τ*_*isc*_ so as to allow the lasing of flavins even at their low physiological intracellular concentrations. We found that lasing flavins would be preferred over NAD(P)H due to its lower threshold requirements, which implies lower risks of inducing damage to cells. We also highlight the benefits of lasing under the two-photon regime, which we recognize to hold more potential for *in vivo* applications than the one-photon regime. In conclusion, we summarize from this theoretical study that lasing unlabelled cells is possible, and can be further developed with novel methods of (i) integrating high quality factor optical cavities with cells; and (ii) minimizing intersystem crossings in fluorophores intracellularly.
